# Vpu Antagonizes BST-2–Mediated Restriction of HIV-1 Release via β-TrCP and Endo-Lysosomal Trafficking

**DOI:** 10.1371/journal.ppat.1000450

**Published:** 2009-05-29

**Authors:** Richard S. Mitchell, Chris Katsura, Mark A. Skasko, Katie Fitzpatrick, David Lau, Autumn Ruiz, Edward B. Stephens, Florence Margottin-Goguet, Richard Benarous, John C. Guatelli

**Affiliations:** 1 Department of Medicine, University of California San Diego, La Jolla, California, United States of America; 2 Department of Anatomy and Cell Biology, University of Kansas Medical Center, Kansas City, Kansas, United States of America; 3 Institut Cochin, Université Paris Descartes, CNRS (UMR 8104), Paris, France; 4 Inserm, U567, Paris, France; 5 CellVir, Genopole, Evry, France; 6 San Diego Veterans Affairs Healthcare System, San Diego, California, United States of America; Northwestern University, United States of America

## Abstract

The interferon-induced transmembrane protein BST-2/CD317 (tetherin) restricts the release of diverse enveloped viruses from infected cells. The HIV-1 accessory protein Vpu antagonizes this restriction by an unknown mechanism that likely involves the down-regulation of BST-2 from the cell surface. Here, we show that the optimal removal of BST-2 from the plasma membrane by Vpu requires the cellular protein β-TrCP, a substrate adaptor for a multi-subunit SCF E3 ubiquitin ligase complex and a known Vpu-interacting protein. β-TrCP is also required for the optimal enhancement of virion-release by Vpu. Mutations in the DSGxxS β-TrCP binding-motif of Vpu impair both the down-regulation of BST-2 and the enhancement of virion-release. Such mutations also confer dominant-negative activity, consistent with a model in which Vpu links BST-2 to β-TrCP. Optimal down-regulation of BST-2 from the cell surface by Vpu also requires the endocytic clathrin adaptor AP-2, although the rate of endocytosis is not increased; these data suggest that Vpu induces post-endocytic membrane trafficking events whose net effect is the removal of BST-2 from the cell surface. In addition to its marked effect on cell-surface levels, Vpu modestly decreases the total cellular levels of BST-2. The decreases in cell-surface and intracellular BST-2 are inhibited by bafilomycin A1, an inhibitor of endosomal acidification; these data suggest that Vpu induces late endosomal targeting and partial degradation of BST-2 in lysosomes. The Vpu-mediated decrease in surface expression is associated with reduced co-localization of BST-2 and the virion protein Gag along the plasma membrane. Together, the data support a model in which Vpu co-opts the β-TrCP/SCF E3 ubiquitin ligase complex to induce endosomal trafficking events that remove BST-2 from its site of action as a virion-tethering factor.

## Author Summary

The cellular protein BST-2 prevents newly formed particles of HIV-1 and other enveloped viruses from escaping the infected cell. HIV-1 encodes the protein Vpu to counteract this host defense, but the mechanism of this antagonism is currently unknown. Here, the data suggest that Vpu recruits the cellular protein β-TrCP to modulate the trafficking of BST-2 within internal cellular membranes, removing BST-2 from its apparent site of action at the cell surface. These results add a new example to the growing paradigm of viral counteraction of so-called “restriction factors,” proteins that provide an innate defense against viruses, by co-option of cellular regulatory assemblies known as multi-subunit ubiquitin ligases.

## Introduction

HIV-1 encodes specific proteins dedicated to counteracting host cell “restriction factors” that inhibit viral replication [1]. In the prototypic example of this relationship, the accessory protein Vif, found in almost all lentiviruses, targets cytidine deaminases in the APOBEC family for proteasomal degradation [2]; these cellular enzymes would otherwise damage nascent viral cDNAs to inhibit infectivity [3]. In the second example of this host-pathogen relationship, the accessory protein Vpu, found almost exclusively in HIV-1 and SIVcpz, counteracts the cellular transmembrane protein BST-2/CD317 (tetherin) [4],[5]. BST-2 is an interferon-induced, cell-surface and lipid-raft associated protein that tethers nascent, fully formed HIV-1 virions to infected cells, preventing their release and subsequent spread [4]–[8]. Vpu decreases the expression of BST-2 at the cell surface [5],[9], and the removal of BST-2 from its site of tethering action may underlie the mechanism by which Vpu counteracts this cellular restriction [5]. However, how Vpu reduces the levels of BST-2 at the cell-surface is currently unknown.

Vpu is a small, transmembrane protein that, in addition to enhancing the release of virions from infected cells [10]–[13], induces the degradation of CD4, and possibly class I MHC, by linking these proteins to the multi-subunit SCF (Skp1-Cullin-F-box)/β-TrCP containing E3 ubiquitin ligase complex [14],[15]. Vpu recruits β-TrCP to membranes of the endoplasmic reticulum to trigger the proteasomal degradation of CD4 [14]. This process requires the interaction of Vpu with β-TrCP [14]. This interaction is mediated by a canonical DpSGxxpS sequence (where pS indicates phosphoserine) in the cytoplasmic domain of Vpu and a propeller-like arrangement of WD repeats in β-TrCP [16],[17]. β-TrCP interacts via its F-box domain with Skp1 and the remainder of the Cullin-1-based E3 ligase complex, leading to the presumed ubiquitination of CD4 and the targeting of CD4 to the proteasome.

The conserved serines in the DpSGxxpS sequence of Vpu are required for the efficient down-regulation of cell-surface BST-2 as well as for the degradation of CD4 [5],[18]. However, Vpu-mediated down-regulation of BST-2 is not effectively blocked by inhibition of the proteasome [5], raising the possibility that Vpu recruits β-TrCP to induce ubiquitin-mediated endosomal trafficking events that remove BST-2 from the cell surface. Furthermore, although the serine residues in the DpSGxxpS sequence contribute to the enhancement of virion-release by Vpu, they are not absolutely required for this activity [5],[18],[19]. This observation has left the role of surface down-regulation in the counteraction of BST-2 by Vpu unresolved.

Here, we show that β-TrCP is required for both the optimal down-regulation of BST-2 and the enhancement of HIV-1 virion-release by Vpu. Vpu-mediated down-regulation of BST-2 from the cell surface is also partly dependent on the plasma membrane associated clathrin adaptor protein complex AP-2 and can be inhibited by disruption of the endo-lysosomal pH gradient. These data suggest that Vpu recruits β-TrCP to induce ubiquitin-mediated endosomal trafficking events that reduce the levels of BST-2 on the plasma membrane, effectively removing BST-2 from its site of action as a virion-tethering factor.

## Results

### β-TrCP is required for optimal down-regulation of BST-2 and enhancement of virion-release by Vpu

**Figure 1:**
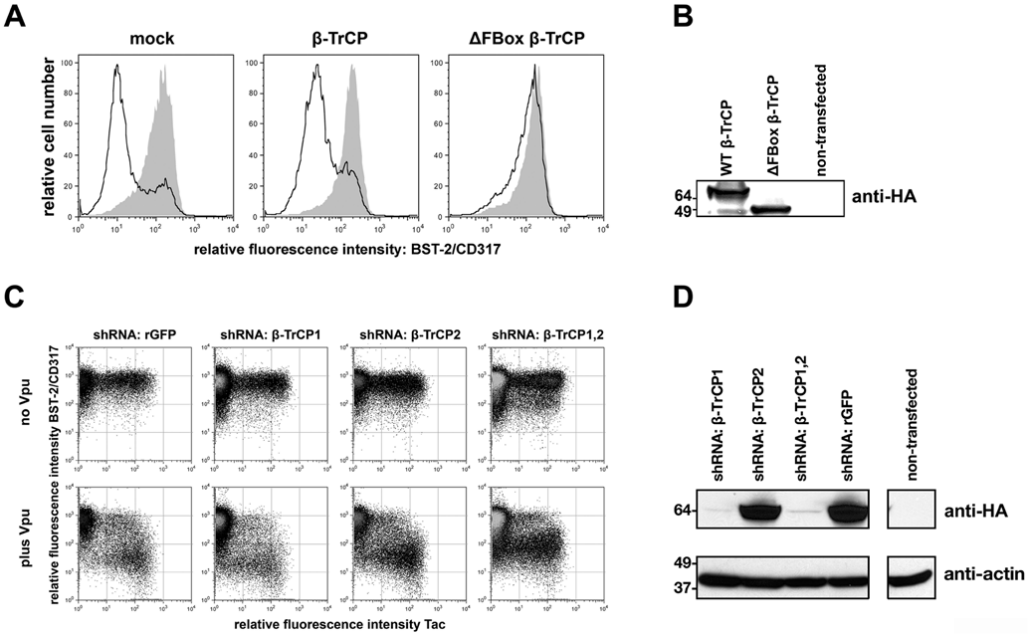
β-TrCP is required for the optimal down-regulation of cell-surface BST-2: inhibition of Vpu activity by ΔF-box β-TrCP and shRNA targeting β-TrCP-1 and -2. (A) ΔF-box β-TrCP inhibits Vpu-mediated down-regulation of cell-surface BST-2. Cells (HeLa) were transfected with either an empty plasmid or a plasmid expressing Vpu, along with a plasmid expressing GFP as a transfection marker. The cells were also transfected with either an empty plasmid (“mock”), a plasmid expressing β-TrCP, or a plasmid expressing a β-TrCP protein lacking the F-box (ΔFbox β-TrCP). The next day, the cells were stained for surface BST-2 and analyzed by two-color flow cytometry. Histograms represent the relative cell number vs. BST-2 fluorescence intensity for the GFP-positive cells. The percentage of GFP-positive cells varied between 30 and 33% for the six transfections shown. Gray-shaded histograms represent cells not transfected to express Vpu; unshaded histograms represent cells transfected to express Vpu. ΔF-box β-TrCP inhibited the Vpu-mediated down-regulation of cell surface BST-2 in each of four experiments; statistical analysis is described in the text. (B) Cells (HeLa) were transfected with a plasmid expressing β-TrCP (WT β-TrCP), or a plasmid expressing a β-TrCP protein lacking the F-box (ΔFbox β-TrCP), or not transfected; cell lysates were analyzed by immunoblot to detect the HA-tagged β-TrCP proteins. (C) shRNA targeting β-TrCP inhibits Vpu-mediated down-regulation of cell-surface BST-2. Cells (HeLa) were transfected with plasmids expressing shRNAs targeting either *Renilla* GFP (rGFP) as an irrelevant control, β-TrCP-1, β-TrCP-2, or both β-TrCP-1 and -2; in two cases these plasmids also expressed jellyfish GFP (the plasmids targeting β-TrCP-1 and both β-TrCP-1 and -2); for the others a separate plasmid expressing GFP was co-transfected. Two days later, the cells were re-transfected with an empty plasmid or a plasmid expressing Vpu, along with a plasmid expressing Tac antigen (IL-2 receptor α; CD25) as a transfection marker. The next day, the cells were stained for surface BST-2 and Tac, and then analyzed by three-color flow cytometry. Two-color dot plots are the BST-2 vs.Tac intensity of the individual GFP-positive cells. The results shown are representative of two independent experiments. (D) HeLa cells were transfected with the indicated plasmids expressing shRNAs used in (C) along with the plasmid expressing β-TrCP-1-HA; cell lysates were analyzed by immunoblot to detect the HA-tagged β-TrCP.

To test the hypothesis that β-TrCP plays a role in the Vpu-mediated down-regulation of BST-2 from the cell surface, we over-expressed a β-TrCP mutant with a deletion in the F-box domain (ΔF-box β-TrCP). β-TrCP binds Vpu via its WD domain, while it binds to the SCF E3 ubiquitin ligase complex via its F box domain. Since ΔF-box β-TrCP binds Vpu but cannot link it and any Vpu-interacting proteins to the ubiquitination machinery, it functions as a dominant negative mutant [14]. As previously reported [5], Vpu down-regulated endogenous BST-2 from the surface of HeLa cells as measured by flow cytometry (Figure 1A, left panel, in which transfection using a Vpu-expression plasmid reduced the mean fluorescence intensity (MFI) of the cells by 69%). Cells transfected to over-express wild-type β-TrCP also supported the efficient down-regulation of BST-2 by Vpu (a reduction of 77% in MFI; Figure 1A, middle panel). In contrast, ΔF-box β-TrCP inhibited the down-regulation of BST-2 by Vpu (a reduction of only 25% in MFI; Figure 1A, right panel). Immunoblot data indicated that the exogenous β-TrCP and ΔF-box β-TrCP proteins were expressed appropriately (Figure 1B). In four independent experiments, the activity of Vpu when wild-type β-TrCP was over-expressed relative to the activity of Vpu alone was 1.10 (standard deviation = 0.22), whereas the activity of Vpu when ΔF-box β-TrCP was expressed relative to the activity of Vpu alone was 0.40 (standard deviation = 0.20); p = 0.007 by Student's t test. The expression of Vpu was not differentially affected in cells expressing ΔF-box β-TrCP relative to cells expressing wild-type β-TrCP, weighing against an indirect mechanism for these effects (data not shown). The inhibition of Vpu-activity by ΔF-box β-TrCP supported key roles for β-TrCP and the linkage of Vpu to the β-TrCP/SCF E3 ubiquitin ligase complex during the down-regulation of BST-2.

To test further the role of β-TrCP in the modulation of BST-2 by Vpu, we used RNA interference to target the endogenous protein. Mammalian cells express two closely related proteins, β-TrCP-1 and β-TrCP-2 [20]. HeLa cells were transfected with plasmids expressing short hairpin RNAs (shRNAs) targeting *Renilla* GFP (as a control), β-TrCP-1, β-TrCP -2, or both β-TrCP-1 and -2; GFP was co-expressed as an indicator of transfection. Subsequently, the cells were re-transfected to express Vpu and a second indicator protein, IL-2 receptor α (Tac antigen; CD25), and then analyzed by flow cytometry for the expression of both indicator proteins and BST-2. The shRNA targeting the sequence common to genes 1 and 2 inhibited the Vpu-mediated down-regulation of BST-2 (Figure 1C); this effect was modest and primarily evident in cells expressing high levels of Tac. In contrast, the shRNAs specific for β-TrCP-1 or -2 had little or no inhibitory activity (Figure 1C). To quantify these effects, comparable regions of peak cell density for the Tac-positive and BST-2 down-regulated cells in each analysis were picked based on contour plots, and the MFI values of these regions were used to determine the fold down-regulation of BST-2 by Vpu in each condition. This analysis indicated that Vpu induced a 17-fold down-regulation of BST-2 in cells expressing the control shRNA targeting Renilla GFP, whereas it induced a 8.5-fold down-regulation in cells expressing the shRNA targeting the sequence common to β-TrCP genes 1 and 2. Using these data plus those from a second, independent experiment (Figure S1), the activity of Vpu in cells expressing shRNA to β-TrCP-1 was 1.39 relative to the activity of Vpu in cells expressing shRNA targeting Renilla GFP, whereas the relative activity in cells expressing shRNA targeting β-TrCP-2 was 0.80, and the relative activity in cells expressing shRNA targeting both β-TrCP-1 and -2 was 0.49. To validate these shRNAs, we tested their effectiveness and specificity using co-expressed, HA-tagged, β-TrCP-1 (Figure 1D). The vector encoding the shRNA specific for β-TrCP-1 and the vector encoding the shRNA targeting both β-TRCP-1 and -2 were equally active against β-TrCP-1-HA, whereas the vector specific for β-TrCP-2 was inactive. These data suggested that targeting β-TrCP-1 alone is insufficient to inhibit the modulation of BST-2 by Vpu. Overall, the inhibitory effect of the shRNA targeting both β-TrCP-1 and -2, which reduced the activity of Vpu by 51% as noted above (see Figure S1) supported the data obtained from the ΔF-box, dominant negative experiments. Both sets of data are consistent with the hypothesis that β-TrCP is a cellular co-factor for the down-regulation of cell-surface BST-2 by Vpu.

**Figure 2:**
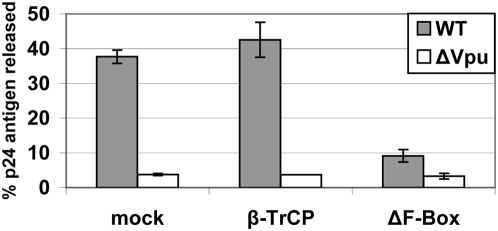
ΔF-box β-TrCP inhibits Vpu-mediated enhancement of virion-release. Cells (HeLa) were transfected with either a proviral plasmid expressing wild-type HIV-1_NL4-3_ (“WT”) or a proviral plasmid expressing an isogenic *vpu*-negative mutant (“ΔVpu”). The cells were also transfected with an empty plasmid (“mock”), a plasmid expressing β-TrCP, or a plasmid expressing the β-TrCP ΔF-Box mutant. The next day, the fraction of the total p24 capsid antigen produced by the cells that was secreted into the media was measured. The average values from two independent experiments are graphed; the error bars indicate the actual values obtained from each experiment.

Since down-regulation from the cell surface has been proposed as the mechanism by which Vpu counteracts the restriction mediated by BST-2 [5], we asked whether over-expression of ΔF-box β-TrCP also inhibited the enhancement of virion-release by Vpu. Vpu-mediated enhancement of virion-release was measured as the fractional secretion of p24 capsid antigen from virus-producing cells. As previously reported [5], Vpu markedly enhanced the efficiency of capsid-release in HeLa cells expressing endogenous BST-2: 38% of the total capsid antigen produced was released into the surrounding medium in the case of cells expressing wild-type virus, whereas only 4% of capsid antigen was released in the absence of Vpu (see “mock” in Figure 2). The over-expression of β-TrCP had little or no effect on these release-efficiencies: 43% for wild-type virus compared to 4% for virus lacking Vpu (Figure 2). In contrast, ΔF-box β-TrCP inhibited the release of wild-type virus, whose efficiency of release was reduced to 9%, while having little or no effect on the release of virus lacking Vpu, whose efficiency of release was 3% (Figure 2). These data indicated that ΔF-box β-TrCP is a selective inhibitor of the release of Vpu-expressing virus, consistent with its ability to inhibit the down-regulation of cell surface BST-2 by Vpu.

ΔF-box β-TrCP inhibits the degradation of CD4 by Vpu [14], and high levels of cell-surface CD4 can inhibit the Vpu-mediated enhancement of virion-release by unclear mechanisms [21]. The HeLa cells used in the preceding experiments express CD4. To determine whether the above results were an indirect consequence of inhibition of CD4-degradation, we repeated the virion-release experiments using a CD4-negative HeLa cell line (Figure S2). Notably, these cells supported a Vpu-phenotype of similar magnitude to that of the CD4-positive cells used throughout this study; Vpu enhanced virion-release by 9.5-fold when CD4-positive HeLa cells were used as viral producers (Figure 2) and by 8.5-fold when CD4-negative HeLa cells were used as viral producers (Figure S2). These data indicated that the levels of CD4 on the HeLa cells used herein were not sufficient to inhibit the enhancement of virion-release by Vpu. Furthermore, the expression of ΔF-box β-TrCP, but not the over-expression of wild-type β-TrCP, inhibited the enhancement of virion-release by Vpu in CD4-negative viral producer cells (Figure S2). Interestingly, the inhibition of Vpu-activity in these experiments was not as great as that observed using the CD4-positive cells, suggesting that a model in which CD4 inhibits release may be operative. Nevertheless, these data indicated that the role of β-TrCP as a co-factor for the enhancement of virion-release by Vpu does not require CD4.

### Residues in the DSGxxS β-TrCP binding motif are required for optimal down-regulation of BST-2 and enhancement of virion-release by Vpu

**Figure 3:**
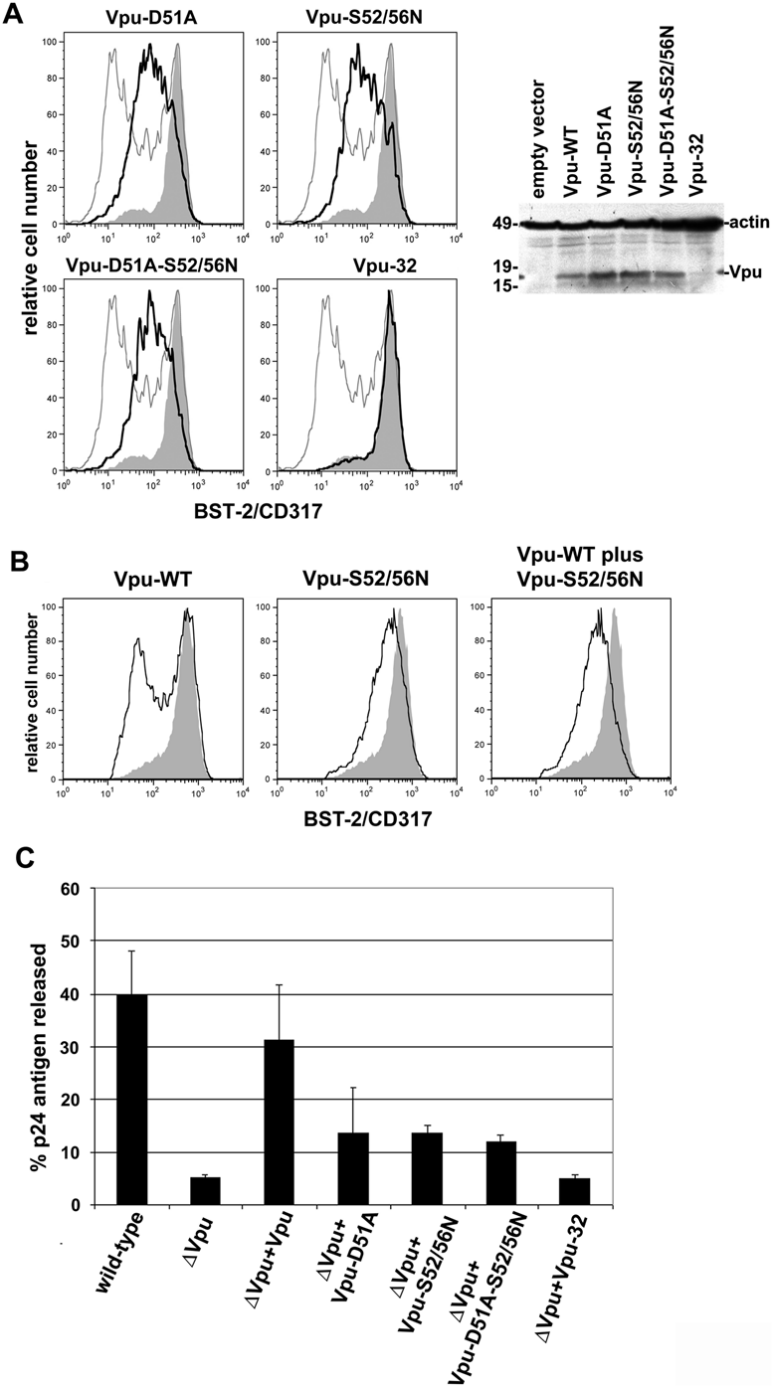
Residues within the DSGxxS β-TrCP binding motif of Vpu are required for optimal down-regulation of BST-2 and enhancement of virion-release. (A) Down-regulation of BST-2 by Vpu-mutants. Cells (HeLa) were transfected with an empty plasmid, a plasmid expressing Vpu, or a plasmid expressing the indicated Vpu mutant, along with a plasmid expressing GFP as a transfection marker. The amount of Vpu-expression plasmid in each transfection (160 ng) was just sufficient in the case of the wild-type to rescue the release of virions from cells expressing a *vpu*-negative genome (see (C) below and the Materials and Methods section). The next day, the cells were stained for surface BST-2 and analyzed by two-color flow cytometry. Left: Histograms represent the relative cell number vs. BST-2 fluorescence intensity for the GFP-positive cells. In each panel, the heavy line is the curve for the indicated Vpu mutant. The shaded curve is the empty vector control, and the light line is the curve for wild-type Vpu. The percentage of cells that were GFP-positive was 11 for Vpu-WT, 14 for Vpu-D51A, 10 for Vpu-S52/56N, and 10 for Vpu-D51A-S52/56N. The results shown are representative of two independent experiments. Right: aliquots of each population were also analyzed by SDS-PAGE and immunoblot for Vpu and actin; molecular weight markers are indicated on the left in kilodaltons. (B) Vpu-S52/56N inhibits down-regulation of BST-2 by the wild-type protein. Cells (HeLa) were transfected as described in (A) above, except that in the right panel a combination of the plasmid expressing wild-type Vpu (160 ng) and the plasmid expressing Vpu-S52/56N (1.0 µg) was used (see also the Materials and Methods section). The next day, the cells were stained for surface BST-2 and analyzed by two-color flow cytometry. Histograms represent the relative cell number vs. BST-2 fluorescence intensity for the GFP-positive cells. The gray-shaded histogram represents cells not transfected to express Vpu (same in each panel); the unshaded histograms represent cells transfected to express Vpu, Vpu-S52/56N, or the combination of WT-Vpu plus Vpu-52/56N. The percentage of GFP-positive cells was 35 for WT-Vpu, 48 for Vpu-52/56N, and 49 for WT-Vpu plus Vpu-52/56N. The results shown are representative of two independent experiments. (C) Enhancement of virion-release by Vpu-mutants. Cells (HeLa) were transfected with a proviral plasmid expressing the *vpu*-negative mutant ΔVpu (1.44 µg), along with a plasmid expressing Vpu or the indicated Vpu mutant (160 ng). For the positive control, cells were transfected with the wild-type proviral plasmid alone; for the negative control, cells were transfected with ΔVpu along with an empty plasmid. The next day, the fraction of the total p24 capsid antigen produced by the cells that was secreted into the media was measured. Results are the average of duplicate transfections and are representative of two independent experiments.

Vpu interacts with β-TrCP via a prototypical DSGxxS motif in its cytoplasmic domain [14]. We previously observed that substitution of these serine residues (52 and 56 in Vpu of HIV-1_NL4-3_) with asparagines markedly inhibited the down-regulation of cell surface BST-2 when Vpu was expressed from a proviral plasmid; this mutation was also associated with a reduction in the efficiency of virion-release [5]. To extend this mutational analysis with respect to the roles of the binding domain for β-TrCP and the cytoplasmic domain of Vpu as a whole, we constructed a Vpu-expression plasmid encoding the serine substitutions, Vpu-S52/56N, as well as three other mutants: Vpu-D51A; Vpu-D51A-S52/56N; and Vpu-32, encoding a truncated Vpu missing most of the cytoplasmic domain including the DSGxxS motif. Vpu-S52/56N and Vpu-D51A were impaired in their ability to down-regulate BST-2 from the cell surface (Figure 3A). This functional impairment was not attributable to poor expression of the mutant proteins at steady state (Figure 3A). Instead, these mutants were expressed at slightly greater levels than the wild-type protein, which may cause an overestimation of their relative activity at the protein level. The phenotype of the combination mutant Vpu-D51A-S52/56N was indistinguishable from that of Vpu-D51A and Vpu-S52/56N, suggesting that the DSGxxS sequence behaves as a single entity and that either the D51A or the S52/56N substitution is sufficient to abolish binding to β-TrCP. The Vpu-32 truncation mutant was devoid of activity, however, the expression of this construct was not verifiable by western blot. Overall, these mutational data supported the role of β-TrCP in the Vpu-mediated down-regulation of BST-2.

To test a model in which Vpu links BST-2 to the β-TrCP/SCF E3 ubiquitin ligase complex (see Figure 1A), we tested the Vpu-S52/56N protein for the ability to dominantly interfere with the activity of wild-type Vpu. The over-expression of Vpu-S52/56N inhibited the down-regulation of BST-2 by wild-type Vpu (Figure 3B). These data suggested that Vpu-S52/56N can saturate endogenous BST-2, prevent wild-type Vpu from linking BST-2 to the β-TrCP/SCF E3 ubiquitin ligase complex, and function as a dominant-negative Vpu-mutant.

Next, we used the mutant panel above to correlate the down-regulation of BST-2 with the enhancement of virion-release. The mutant Vpu proteins were tested for their ability to rescue the efficiency of virion-release when expressed in *trans* (Figure 3C). To avoid misinterpretation due to over- or under-expression, the amount of plasmid expressing wild-type Vpu was titrated to the minimum required for *trans*-complementation (data not shown; this amount of plasmid was also used for all the Vpu-expression constructs in the experiment of Figure 3A and in the experiment of Figure 3B for wild-type Vpu). Vpu-S52/56N, Vpu-D51A, and Vpu-D51A-S52/56N were impaired in their ability to enhance virion release, whereas Vpu-32 was completely inactive. These mutational data confirmed the role of the β-TrCP binding motif in the enhancement of virion-release by Vpu, and they supported a correlation between the ability of Vpu to enhance virion-release and its ability to down-regulate cell-surface BST-2.

### Vpu induces a modest reduction in the total cellular levels of BST-2

**Figure 4:**
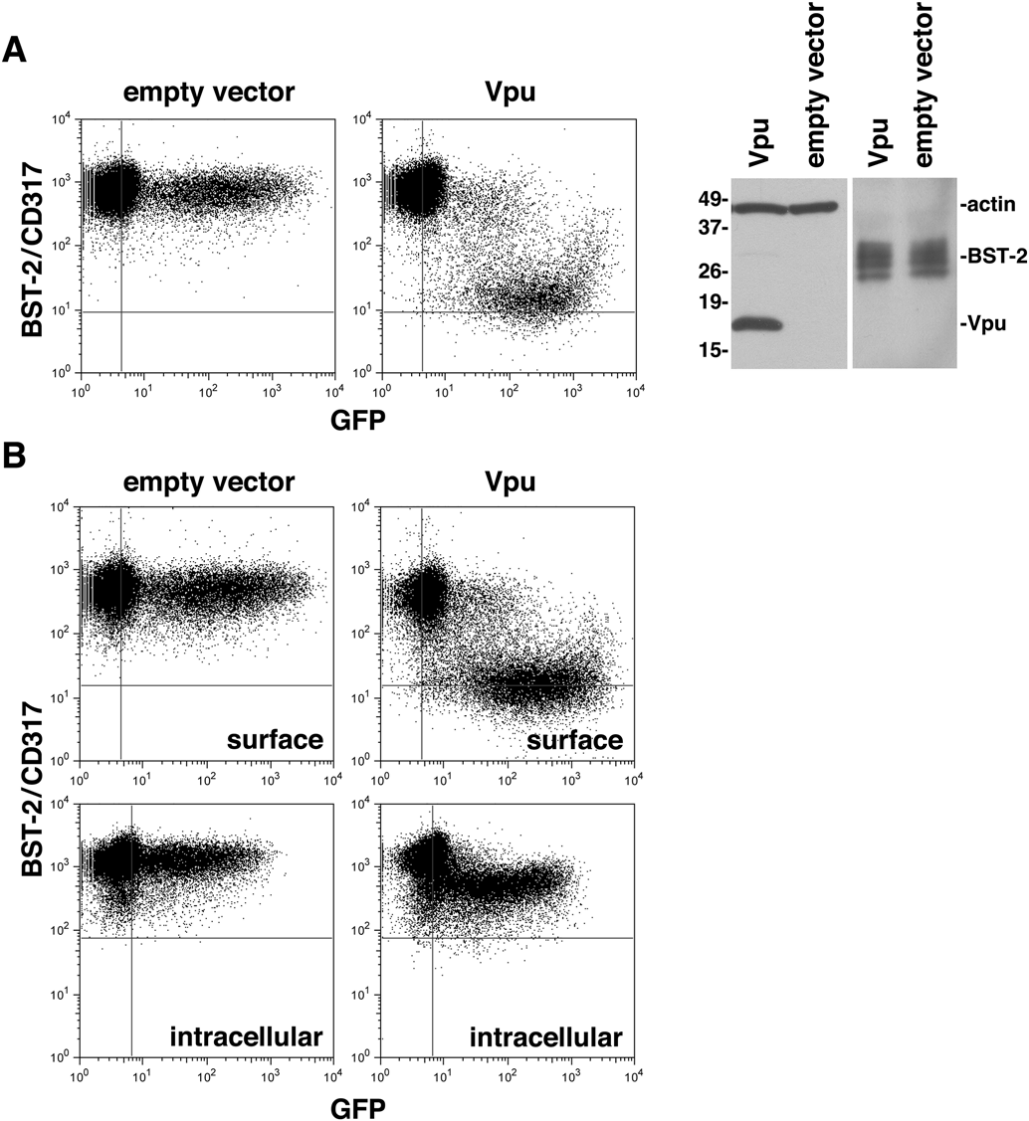
Vpu decreases total cellular BST-2 to a lesser extent than cell-surface BST-2. (A) Effect of Vpu on the steady-state total cellular levels of BST-2 detected by immunoblot. Cells (HeLa) were transfected with an empty plasmid or a plasmid expressing Vpu along with a plasmid expressing GFP as a transfection marker in a 20∶1 weight ratio. Left: The next day, the cells were stained for surface BST-2 and analyzed by two-color flow cytometry: two-color dot plots are the BST-2 vs. GFP intensity of the individual cells. Right: aliquots of each population were also analyzed by SDS-PAGE and immunoblot for Vpu, actin and for BST-2; molecular weight markers are indicated on the left in kilodaltons. (B) Effect of Vpu on intracellular and surface levels of BST-2 measured by flow cytometry. Cells (HeLa) were transfected as described in (A) with an empty plasmid or a plasmid expressing Vpu along with a plasmid expressing GFP as a transfection marker. The next day, the cells were stained for BST-2 either without (“surface”) or with (“intracellular”) permeabilization and analyzed by two-color flow cytometry: two-color plots are the BST-2 vs. GFP intensity of the individual cells.

Since a β-TrCP-mediated mechanism of down-regulation could involve a reduction in the total cellular levels of BST-2 via ubiquitin-mediated proteasomal and/or endo-lysosomal degradation, we sought to measure the effect of Vpu on the total cellular levels of BST-2. Under conditions in which a robust down-regulation of BST-2 from the cell surface was detected by flow cytometry, no effect on the total steady-state levels of BST-2 protein was detected by immunoblot (Figure 4A, which reveals multiple immunoreactive bands in the range of 25–36 kilodaltons consistent with heterogeneous glycosylation of BST-2 [22]). Because this analysis was potentially limited by the transfection efficiency (36% GFP-positive cells in the transfection including the Vpu-expression vector in Figure 4A), we used intracellular staining and flow cytometry to quantify total cellular BST-2 in transfected cells and compared this to cell-surface levels. While the levels of BST-2 on the cell surface were reduced 10-fold by Vpu, the total cellular levels were reduced by only 1.8 fold. A very subtle effect of Vpu on the total cellular expression of BST-2 was observed when cells from a separate experiment were physically sorted to enrich for GFP-expression prior to analysis by immunoblot (Figure S3), and when BST-2 was expressed by transient transfection together with Vpu in HEK 293 T cells (data not shown). Together, these data suggested that Vpu modestly decreases the total cellular levels of BST-2, consistent with previous data [9], although whether this decrease is sufficient to account for the reduction in the level of BST-2 on the cell surface is unclear.

### The plasma membrane clathrin adaptor protein complex AP-2 is required for optimal down-regulation of cell surface BST-2 by Vpu

**Figure 5:**
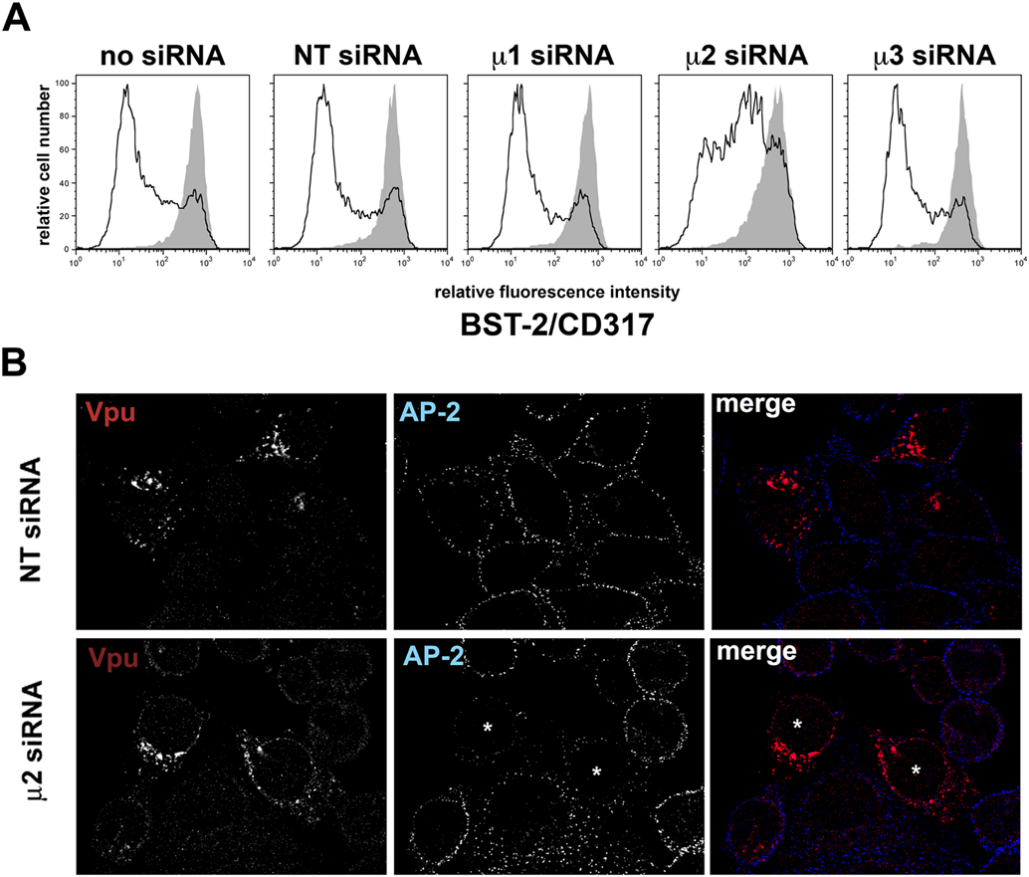
The plasma membrane clathrin adaptor protein complex AP-2 is required for optimal down-regulation of BST-2 from the cell surface by Vpu. (A) Cells (HeLa) were transfected once with siRNAs targeting either the medium (μ) subunits of AP-1 (μ1), AP-2 (μ2), AP-3 (μ3), or an irrelevant “non-target” (NT) sequence. Three days later, the cells were re-transfected with either an empty plasmid or a plasmid expressing Vpu, along with a plasmid expressing GFP as a transfection marker. The next day, the cells were stained for surface BST-2 and analyzed by two-color flow cytometry. Histograms represent the relative cell number vs. BST-2 fluorescence intensity for the GFP-positive cells. Gray-shaded histograms represent cells not transfected to express Vpu; unshaded histograms represent cells transfected to express Vpu. Inhibition of Vpu-mediated down-regulation of BST-2 by siRNA targeting μ2 was observed in each of four independent experiments. (B) Cells (HeLa) were transfected once with siRNAs targeting either μ2 or an irrelevant “non-target” (NT) sequence. Three days later, the cells were re-transfected with a plasmid expressing Vpu. The next day, the cells were fixed, permeabilized, and stained for Vpu and AP-2. The cells were imaged as described in the Materials and Methods section; a single focal plane is shown. Asterisks indicate cells with reduced expression of AP-2.

Because Vpu reduced the level of BST-2 on the cell surface more dramatically than it reduced the total cellular level of BST-2, we considered a model in which Vpu and the β-TrCP/SCF E3 ubiquitin ligase complex remove BST-2 from the plasma membrane via an influence on ubiquitin-mediated endosomal trafficking [23],[24]. Ubiquitination is a regulatory mechanism of endocytosis (reviewed in [25]), and in some examples this requires the plasma membrane-associated clathrin adaptor AP-2 [26]. AP-2 is a member of the endosomal adaptor protein (AP) complex family. These heterotetrameric complexes coat endosomal membranes, where they recruit cargo proteins and in some cases the vesicle-scaffolding protein clathrin (reviewed in [27]). To test the role of AP complexes in the down-regulation of BST-2 by Vpu, we used siRNA to target the medium (μ) subunits of three of the four members of the AP complex family. siRNA targeting the μ subunit of AP-2 (μ2), but not siRNAs targeting the μ subunits of AP-1 (μ1) or AP-3 (μ3), inhibited the Vpu-mediated down-regulation of BST-2 (Figure 5A). This inhibition was partial, reflecting either an incomplete knockdown [supported by immunoblot data (not shown) and immunofluorescence microscopy (Figure 5B)] or additional mechanisms of down-regulation. Notably, the knockdown of AP-2 had no effect on the surface levels of BST-2 in the absence of Vpu.

To exclude that the knockdown of AP-2 acted on Vpu rather than BST-2, we examined the distribution of Vpu in cells treated with siRNA to μ2 by immunofluorescence microscopy (Figure 5B). The knockdown of AP-2 was incomplete under these conditions, with at most half the cells showing reduced expression of the α subunit of the complex. As noted previously [28], the distribution of Vpu (here from HIV-1_NL4-3_, a group M, clade B genome) was endosomal, and steady-state expression at the plasma membrane was minimal. Notably, little co-localization of Vpu and AP-2 was observed. Furthermore, regardless of the levels of AP-2, Vpu maintained an endosomal distribution; it was not displaced to the plasma membrane by knockdown of AP-2. These data weighed against the possibility that the knockdown of AP-2 affected Vpu trafficking and consequently function. Instead, the data suggested that AP-2 mediates the trafficking of BST-2 in a manner that supports down-regulation by Vpu.

### The action of Vpu is post-endocytic and partly dependent on the endo-lysosomal pH gradient

**Figure 6:**
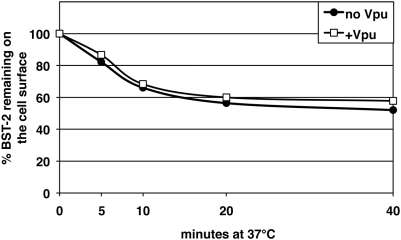
Vpu does not increase the rate of endocytosis of BST-2. Cells (HeLa) were transfected with either an empty plasmid (“no Vpu”) or a plasmid expressing Vpu (“plus Vpu”), along with a plasmid expressing GFP as a transfection marker. The next day, the cells were labeled at 4°C with an antibody to BST-2, warmed for the indicated times at 37°C, then stained with a fluorophore-conjugated secondary antibody and analyzed by two-color flow cytometry. The amount of BST-2 remaining on the cell surface over time is shown for the GFP-positive cells. The fluorescence intensities of the time zero cells (no incubation at 37°C) for each population (“no Vpu” and “plus Vpu”) were set at 100%. The expression of Vpu reduced the surface levels of BST-2 by 3-fold in this experiment (data not shown). The results shown are representative of two independent experiments.

The simplest explanation for the preceding data is that Vpu recruits β-TrCP to induce ubiquitin-mediated endocytosis of BST-2. To test this, we compared the rate of endocytosis of BST-2 in cells that were expressing Vpu with those that were not (Figure 6). Human BST-2 was internalized constitutively, consistent with previous studies of rodent BST-2 [29]. Surprisingly, although Vpu down-regulated the steady-state expression of BST-2 on the cell surface (data not shown), the fractional rate of internalization of BST-2 from the cell surface was unaffected. These data suggested that although AP-2 and presumably the endocytosis of BST-2 are required for efficient down-regulation, the actual site of action of Vpu is post-endocytic.

**Figure 7:**
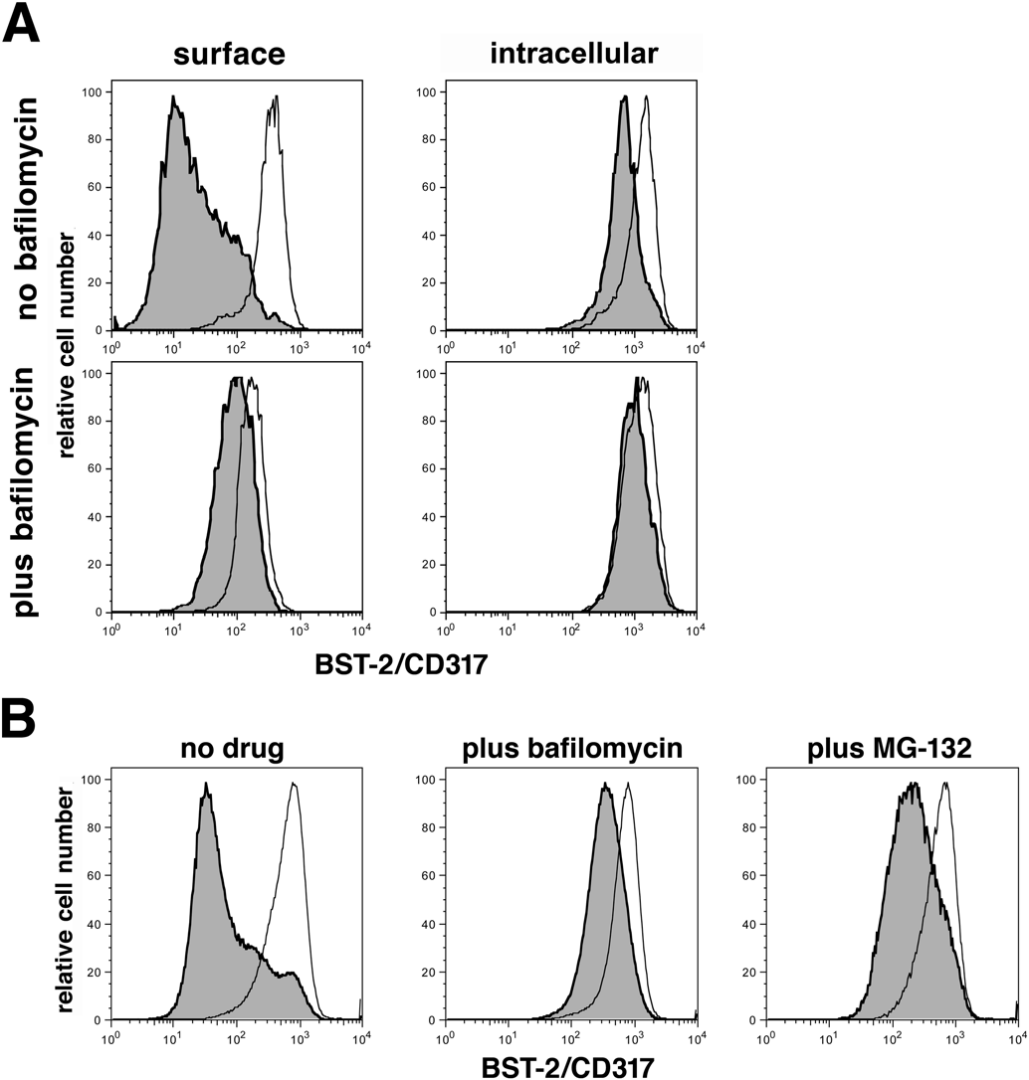
Bafilomycin A1 inhibits the ability of Vpu to down-regulate BST-2. (A) Cells (HeLa) were transfected with either an empty plasmid or a plasmid expressing Vpu, along with a plasmid expressing GFP as a transfection marker. Immediately after the transfection, the cells were treated with bafilomycin A1 (final concentration 0.13 µM in DMSO) or DMSO only for 14 hours, and then stained for surface or intracellular BST-2 and analyzed by two-color flow cytometry. Histograms represent the relative cell number vs. BST-2 fluorescence intensity for the GFP-positive cells. Gray-shaded histograms represent cells transfected to express Vpu; unshaded histograms represent cells transfected with the empty plasmid. The plots shown are representative of at least two transfections for each experimental condition. (B) Cells (HeLa) were transfected as described above. Immediately after the transfection, the cells were treated with bafilomycin A1 (final concentration 0.13 µM in DMSO), MG-132 (final concentration 30 µM in DMSO), or DMSO only for 14 hours, and then stained for surface BST-2 and analyzed by two-color flow cytometry. Histograms represent the relative cell number vs. BST-2 fluorescence intensity for the GFP-positive cells. Gray-shaded histograms represent cells transfected to express Vpu; unshaded histograms represent cells transfected with the empty plasmid. The plots shown are representative of two independent experiments.

To test the hypothesis that Vpu influences BST-2 at a post-endocytic trafficking step, we examined the effect of the endosomal proton-pump inhibitor bafilomycin A1 on the down-regulation of BST-2. By blocking the activity of the endosomal vATPase, bafilomycin A1 inhibits acidification of the endosomal system, which normally maintains a gradient of decreasing pH from early/sorting endosomes to late endosomes and lysosomes. Consequently, bafilomycin A1 has at least two effects: it inhibits pH-gradient dependent trafficking to late endosomes and lysosomes [30]–[32], and it inhibits acid-dependent lysosomal degradation [33]. Here, bafilomycin A1 inhibited the down-regulation of BST-2 from the cell surface by Vpu (Figure 7A). Bafilomycin A1 also inhibited the reduction of intracellular BST-2 induced by Vpu. Notably, in some experiments, bafilomycin A1 appeared to induce a modest decrease in the level of cell-surface (but not intracellular) BST-2 independently of Vpu; this effect could be due to its ability to delay transport along the biosynthetic, exocytic pathway [34]. Bafilomycin A1 did not affect the steady-state expression of Vpu itself (data not shown). These data on the inhibitory effect of bafilomycin A1, together with the data on endocytic rate and the role of AP-2, suggested that Vpu acts at a post-endocytic step to reduce the cell surface levels of BST-2. The data are consistent with a model in which Vpu targets BST-2 that has been endocytosed constitutively via AP-2 to late endosomes and lysosomes, where it is sequestered from the plasma membrane and partially degraded.

We previously reported that the treatment of cells expressing Vpu with the proteasome inhibitor MG-132 for five hours did not effectively restore BST-2 to the cell surface, leading to the conclusion that surface down-regulation is not a direct consequence of proteasomal degradation [5]. Here, to test the hypothesis that the down-regulation of BST-2 from the cell surface is ubiquitin-dependent, we treated Vpu-expressing cells with MG-132 for 14-hours, reasoning that this longer duration of proteasome-inhibition was more likely capable of reducing the cellular pools of free ubiquitin. Prolonged treatment with MG-132 inhibited the Vpu-mediated down-regulation of BST-2, although not as effectively as treatment with bafilomycin A1 (Figure 7B). These data supported the hypothesis that the down-regulation of BST-2 by Vpu is at least in part ubiquitin-dependent.

### Vpu-mediated surface down-regulation reduces the co-localization of BST-2 and the virion proteins p17/p55 Gag along the plasma membrane of virus-producing cells

**Figure 8:**
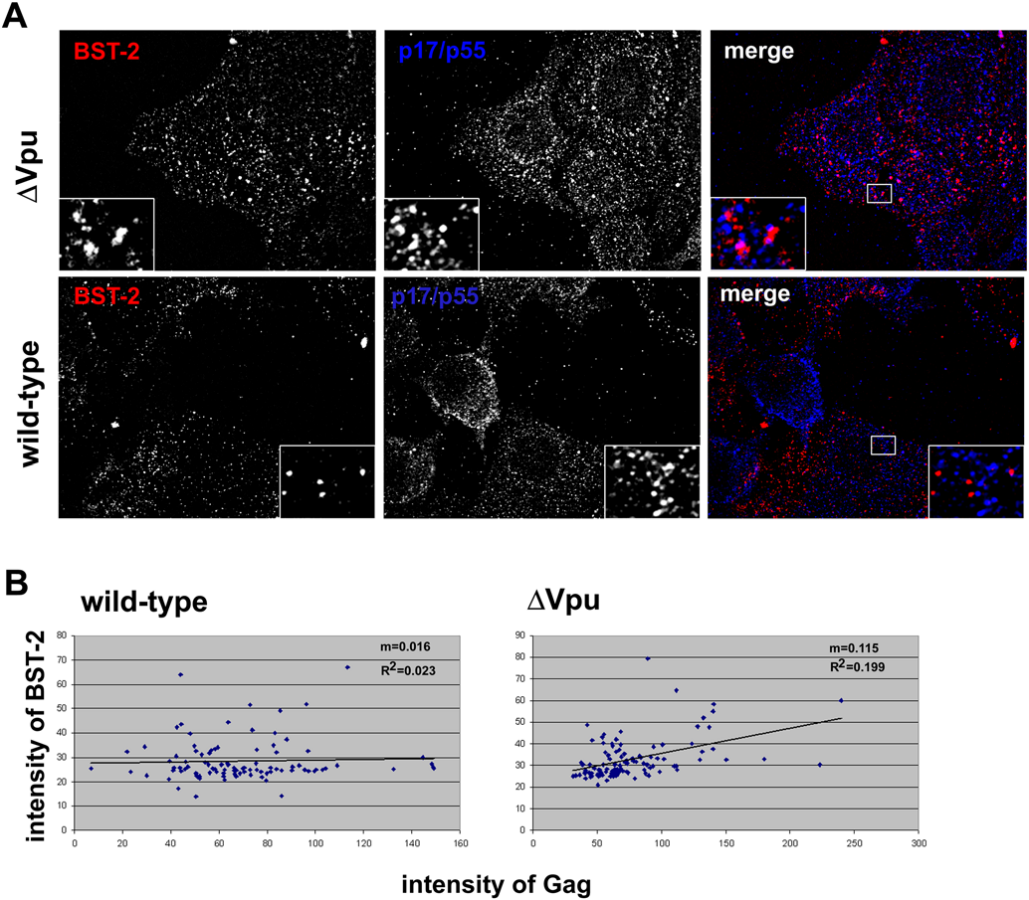
Vpu decreases the co-localization of BST-2 and the virion-protein p17 Gag along the plasma membrane. (A) Cells (HeLa) were transfected to express either wild-type (*vpu*-positive) or *vpu*-negative (ΔVpu) viral genomes. The next day, the cells were fixed and stained without permeabilization for surface BST-2 (red). The cells were subsequently permeabilized with detergent and stained for p17/p55 Gag (blue). Images were acquired in a focal plane just above the cover glass to capture the distribution of proteins along the plasma membrane. (B) Correlation of the relative staining intensities of BST-2 and Gag in cell surface puncta; units are arbitrary.

The mutual dependence of the down-regulation of BST-2 and the enhancement of virion-release on β-TrCP suggests a causal relationship between these two effects of Vpu. The idea that Vpu counteracts restriction of virion-release by removing BST-2 from the cell surface seems intuitively obvious, but modulation of the subcellular distribution of BST-2 by Vpu was not evident in initially published microscopic data [4],[5]. These studies examined the expression of BST-2 in optical sections of permeabilized cells. We reevaluated this paradox by specifically imaging the cell surface, both by staining the cells for BST-2 before permeabilization and by acquiring images just above the cover glass (Figure 8). These data revealed that the surface of cells expressing endogenous BST-2 and *vpu*-negative virus is covered with a punctate distribution of both BST-2 and the viral structural protein p17/p55 Gag (Figure 8A). In many cases these puncta overlapped and likely represented the co-localization of BST-2 and Gag in endocytic pits and/or at sites of virion assembly. In contrast, in cells expressing wild-type (*vpu*-positive) virus, BST-2-containing puncta were diminished in intensity or completely absent, and less co-localization of BST-2 and Gag was detected. These images are similar to those of cells transiently expressing epitope-tagged BST-2 [35], although the co-localization between transiently expressed BST-2 and Gag in the absence of Vpu appears even more striking than that shown here for endogenous BST-2; this difference could be due to supra-physiologic expression following transient transfection and/or to masking of the native epitope by bound virions. Here, analysis of over 10 cells expressing either wild-type or *vpu*-negative virus indicated an average Pearson coefficient of correlation between surface BST-2 and Gag signals of 0.048 for cells expressing the wild-type and 0.11 for cells expressing *vpu*-negative virus (1.0 indicates a perfect positive correlation; 0 indicates no correlation; and −1.0 indicates a perfect negative correlation); p = 0.002 by Student's t test (data not shown). Analysis of over 100 individual surface puncta picked randomly in the Gag image-channel on seven cells expressing virus of each genotype indicated no relationship between the intensity of BST-2 and Gag in the case of wild-type but a trend toward a positive correlation in the case of *vpu*-negative virus (Figure 8B). These data indicated that the down-regulation of BST-2 from the cell surface by Vpu occurs during viral gene expression in a manner consistent with counteraction of virion-tethering activity.

## Discussion

**Figure 9:**
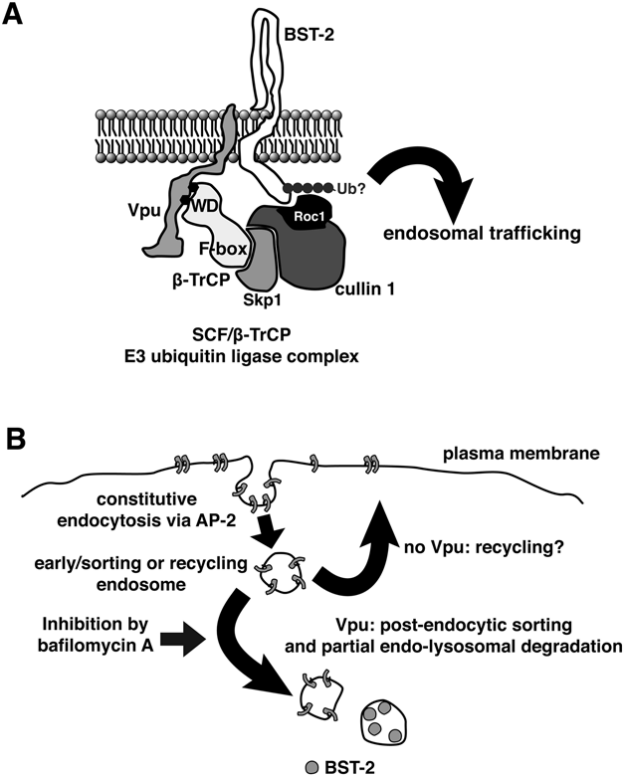
Model for the relief of BST-2-mediated restriction by Vpu. (A) Vpu recruits β-TrCP to induce ubiquitin-mediated trafficking events that remove BST-2 from the plasma membrane, its site of action as a virion-tethering factor. Circles in the cytoplasmic domain of Vpu represent phosphoserines 52 and 56. The interaction between BST-2 and Vpu and the ubiquitination of BST-2 are currently speculative. (B) Vpu induces bafilomycin A1-sensitive post-endocytic trafficking of BST-2 and endo-lysosomal degradation. The removal of BST-2 from the plasma membrane involves constitutive endocytosis of BST-2 via AP-2, followed by Vpu-mediated post-endocytic sorting events. Recycling of BST-2 to the plasma membrane in the absence of Vpu is currently speculative.

The data herein indicate that the Vpu-mediated removal of the transmembrane protein BST-2/CD317 (tetherin) from the cell surface and the Vpu-mediated enhancement of virion release are correlated by a mutual dependence on the cellular co-factor β-TrCP, a substrate adaptor for an SCF E3 ubiquitin ligase complex. β-TrCP/SCF E3 ligase complexes recognize numerous physiologic cellular substrates, targeting some for ubiquitin-mediated proteasomal degradation and others for ubiquitin-mediated endocytosis [14], [23], [24], [36]–[38]. We propose that Vpu recruits β-TrCP to remove BST-2 from the cell surface via post-endocytic membrane trafficking events (Figure 9). The consequence is reduced expression of BST-2 at the plasma membrane, decreased availability of BST-2 for interaction with nascent virions, and counteraction of the ability of BST-2 to restrict the release of progeny virions from infected cells.

The conclusion that β-TrCP plays a role in the Vpu-mediated modulation of BST-2 and the relief of restriction derives from three experimental approaches: expression of a dominant negative mutant of β-TrCP, expression of shRNAs targeting β-TrCP, and functional characterization of conserved residues within the binding site on Vpu for β-TrCP. Each of these experimental approaches inhibited the down-regulation of cell surface BST-2 and/or the enhancement of virion-release, but in each case the inhibition was incomplete. These results suggest that the recruitment of the β-TrCP/SCF E3 ligase complex enables optimal Vpu-activity but is not frankly obligatory. β-TrCP-independent activity could rely on direct binding between Vpu and BST-2 and endosomal sequestration as discussed below. Interestingly, the expression of an shRNA targeting β-TrCP-1 and -2 inhibited Vpu-activity less efficiently than the expression of the dominant negative mutant, Δ-F-box β-TrCP. We suspect this difference may be a technical one. Alternatively, the activity of the Δ-F-box mutant could in principle be due to competition with a WD- and F-box containing protein other than β-TrCP-1 or -2, but we consider this very unlikely, because over-expression of full-length β-TrCP-1 did not inhibit Vpu-activity.

The proposed model for the down-regulation of BST-2 by Vpu from the cell surface (Figure 9) incorporates three key components: β-TrCP and by inference ubiquitination, the plasma membrane clathrin adaptor AP-2; and endosomal acidification. The proposed itinerary for BST-2 is internalization from the plasma membrane via AP-2, then sorting by Vpu/β-TrCP-mediated ubiquitination followed by pH-dependent endosomal transport. Alternative models are possible. Specifically, the roles of AP-2, β-TrCP-mediated ubiquitination, and endosomal acidification could represent independent mechanisms of action of Vpu. Nevertheless, the model proposed is attractive because it incorporates all the data into a single pathway. The model is also consistent with the physiologic roles of β-TrCP in the removal of cellular receptors from the plasma membrane. These cellular transmembrane proteins, which include the human growth hormone, prolactin, and the type-1 interferon receptors, are down-regulated from the cell surface by β-TrCP-dependent endocytosis and/or lysosomal degradation [23],[24],[38],[39].

The data herein indicate that in addition to modulating cell-surface levels, Vpu modestly decreases the total cellular concentration of BST-2. Both the decrease in surface levels and the decrease in intracellular BST-2 were sensitive to the endosomal proton pump (vATPase) inhibitor bafilomycin A1. These data suggest that Vpu induces, at least to some extent, the lysosomal degradation of BST-2. However, we suspect on quantitative grounds that lysosomal degradation is a consequence of the removal of BST-2 from the cell surface rather than the cause of the reduced surface levels. Given the relatively modest extent of degradation, we speculate that a substantial fraction if not the majority of the surface down-regulation effect results from the sequestration of BST-2 within endosomes. Intriguingly, the virally encoded RING-CH ubiquitin ligase K5 of KHSV, which like Vpu down-regulates BST-2, uses a mechanism similar to that just proposed to remove the natural killer cell ligand MICA from the cell surface: targeting surface MICA to an endosomal compartment without inducing marked degradation [40].

Notably, a more striking decrease in the total cellular expression of BST-2 than is shown herein was previously observed during the expression of Vpu in HeLa cells and macrophages, though apparently not in certain T cell lines [9],[41]. These quantitative differences may relate to differing levels of expression, but how Vpu would antagonize BST-2 in the absence of either down-regulation from the cell surface and/or a decrease in total cellular expression is unclear [41]. Of further interest, treatment of cells with proteasome-inhibitors was very recently shown to reverse a Vpu-induced decrease in the total cellular expression of exogenous, epitope-tagged BST-2 in HEK293 cells [42]. These data led to the proposal that Vpu induces the proteasomal degradation of BST-2. Notably, these experiments involved prolonged incubation of cells with the proteasome-inhibitors, which likely depletes the cellular pool of free ubiquitin [43]. Thus, a more inclusive interpretation of such results is that depletion of cellular BST-2 by Vpu is ubiquitin-dependent. This conclusion is mechanistically consistent with the data herein, insofar as ubiquitin-dependent processes include not only proteasomal degradation but also endosomal trafficking and lysosomal degradation. We previously reported that the exposure of HeLa cells to the proteasome inhibitor MG-132 for five hours minimally reversed the Vpu-mediated down-regulation of surface BST-2 [5], but here we show that a 14-hour exposure induces a more striking inhibition. These data support the hypothesis that the effect of proteasome-inhibitors on the Vpu-induced down-regulation of BST-2 reflects ubiquitin-depletion. Overall, although the prolonged exposure to proteasome-inhibitors cannot distinguish between proteasomal degradation and other ubiquitin-dependent mechanisms, the roles of AP-2 and β-TrCP together with the inhibition by bafilomycin A1 reported here support a model of ubiquitin-mediated endo-lysosomal trafficking.

As noted above, the plasma membrane adaptor protein complex AP-2 was required for the efficient down-regulation of BST-2 by Vpu. This result is consistent with the observation that rodent BST-2, though associated with lipid rafts via its C-terminal GPI-anchor, is internalized constitutively via the clathrin adaptor AP-2 [29]. This result also leads to the conclusion that at least part of the down-regulation results from the direct removal of BST-2 from the plasma membrane, a mechanism that may provide the most rapid reduction of cell surface levels. Alternative mechanisms such as total cellular depletion or retention of BST-2 within biosynthetic or exocytic membrane systems rely on the physiologic rate of turnover at the plasma membrane to clear BST-2 from its site of virion-tethering action. Since Vpu is expressed late during the viral life cycle concurrently with the structural proteins of the virion, the direct removal of BST-2 from the plasma membrane via endosomal trafficking could be key to the temporally effective relief of restricted virion-release.

Exactly how the Vpu-mediated recruitment of β-TrCP would affect the endosomal trafficking of BST-2 remains to be elucidated. The rates of endocytosis of BST-2 in the presence and absence of Vpu were equivalent, an observation that weighs against the involvement of proteins such as epsin and eps15, which participate in endocytosis and which recognize ubiquitin as a sorting signal [26],[44]. Instead, the influence of Vpu on BST-2 is apparently post-endocytic. This conclusion is based both on the absence of an effect of Vpu on endocytosis and on the inhibition of down-regulation obtained by disruption of the endosomal pH gradient by treatment of cells with bafilomycin A1. We favor the hypothesis that Vpu and the β-TrCP/SCF E3 ligase complex influence the itinerary of BST-2 at the level of early, sorting endosomes. Here, we speculate that Vpu diverts BST-2 from a recycling pathway, which would normally return it to the cell surface, toward late endosomes, in which BST-2 would become sequestered and partially degraded (Figure 9B). Candidate ubiquitin interacting proteins that could mediate such a sorting event include the monomeric clathrin adaptor Hrs, which plays a canonical role in recruiting endocytosed epidermal growth factor receptor for transport to late endosomes at this junction within the endosomal system [45].

Another key question that emerges from our model is whether or not Vpu induces the ubiquitination of BST-2. Notably, we have observed that mutation of both lysines in the cytoplasmic domain of BST-2 does not block the down-regulation of surface expression by Vpu (data not shown). These data suggest that if Vpu and the β-TrCP/E3 ligase complex induce ubiquitination of BST-2, then this must occur on non-lysine residues. Nevertheless, ubiquitination of BST-2 itself is not an essential feature of a model involving β-TrCP; for example, the requirement for β-TrCP in the internalization of growth hormone receptor (GHR) appears to be independent of GHR ubiquitination [24].

The model herein includes the hypothesis that Vpu and BST-2 interact (Figure 9A). While this remains to be demonstrated, endosomal co-localization of Vpu and BST-2 has been observed at the level of light microscopy [4],[5]. Intriguingly, robust co-localization required the native sequence of the Vpu transmembrane domain. Furthermore, the transmembrane domain (TMD) of Vpu is required for both the down-regulation of surface BST-2 and the enhancement of virion-release [5],[19]. Consequently, it is tempting to speculate that Vpu and BST-2 interact via their transmembrane domains. This hypothesis is supported by our observations that BST-2 from rhesus macaques, which is not efficiently down-regulated from the cell surface by Vpu, is rendered partly Vpu-responsive by the introduction of mutations in the BST-2 TMD that “humanize” the sequence (data not shown). It is also supported by recent data indicating that the inability of Vpu to antagonize the restrictive effect of rhesus BST-2 is a consequence of amino acid changes in the TMD of the rhesus relative to the human protein [46]. Here, the mutated protein Vpu-S52/56N dominantly interfered with the down-regulation of BST-2 by wild-type Vpu. This result is consistent with a model in which a ternary interaction enables Vpu to link BST-2 to β-TrCP. Presumably, Vpu-S52/56N can bind BST-2 via its transmembrane domain but cannot link it to β-TrCP. Consequently, the over-expression of Vpu-S52/56N may saturate BST-2 and competitively inhibit the activity of the wild-type Vpu protein.

As shown here and elsewhere, mutation of the DSGxxS sequence impairs virion-release, but it does not abolish this activity [5],[18]. Conversely, a Vpu truncation mutant including a minimal cytoplasmic domain that does not contain the DSGxxS sequence reportedly retained partial activity in enhancing virion-release [19]. To reconcile these observations with the model proposed here, we speculate that a minimal Vpu lacking most of the cytoplasmic domain or a Vpu lacking a functional β-TrCP binding motif may retain a modicum of activity in counteracting BST-2, solely on the basis of its ability to interact with BST-2 and trap it within the endosomal system. Nevertheless, the data herein indicate that the recruitment of the β-TrCP/SCF E3 ubiquitin ligase complex by the full-length, wild-type Vpu protein enables more efficient removal of BST-2 from the cell surface and consequently more efficient enhancement of virion-release.

A remarkable feature of this emerging model is that it unites the transmembrane and cytoplasmic domains of Vpu in the context of counteracting the restriction imposed by BST-2. This is a new perspective on the Vpu protein, whose domains have been previously associated with “separable” functions: the TMD with the enhancement of virion-release and the cytoplasmic domain with the degradation of CD4 [19]. Indeed, the cytoplasmic domain of Vpu is sufficient to induce the degradation of CD4 in the absence of the native Vpu TMD sequence, because it contains the determinants for both the interaction with CD4 and the recruitment of β-TrCP [14],[47]. In contrast, both the transmembrane and the cytoplasmic domains of Vpu contribute to the enhancement of virion-release [5],[18],[19], in accordance with the model proposed herein.

The spectrum of viruses restricted by BST-2 and the nature of viral countermeasures to this host defense are currently emerging. To date, BST-2 has been shown to restrict the release of members of the retrovirus, filovirus, and arenavirus families. Viral antagonists of BST-2 include, in addition to Vpu, the K5 protein of KSHV (HHV-8), the envelope glycoprotein of HIV-2, and the envelope glycoprotein of Ebola virus [9],[35],[48],[49]. As noted above, the K5 and Vpu proteins share mechanistic features, insofar as K5 is a membrane-associated ubiquitin ligase, and Vpu recruits a cellular ubiquitin ligase complex to membranes. How viral envelope glycoproteins antagonize BST-2 is unclear, but the HIV-2 envelope down-regulates BST-2 from the cell surface, and this requires the tyrosine-based AP-2 binding motif in the cytoplasmic domain of gp41 (data not shown). So far, viral antagonists of BST-2 appear to operate via the cellular ubiquitination and/or endosomal trafficking systems.

In summary, we show that β-TrCP is a Vpu-cofactor for the removal of BST-2 from the plasma membrane and consequently for the counteraction of the antiviral activity of this interferon-induced restriction factor. The data herein support a model in which Vpu utilizes the β-TrCP/E3 ubiquitin ligase complex to modulate the itinerary of BST-2 within the endosomal system via a post-endocytic mechanism. Although the molecular details of how the recruitment of the β-TrCP/E3 ubiquitin ligase complex affects the trafficking of BST-2 remain to be elucidated, these data add a new mechanism to the growing paradigm of viral counteraction of cellular restriction factors by co-option of cellular multi-subunit ubiquitin ligase complexes.

## Materials and Methods

### Plasmids, antibodies, and reagents

pcDNA3.1 (Invitrogen, Carlsbad, CA) was used as an empty vector control. Plasmids expressing HA-tagged β-TrCP-1 and ΔF-box b-TrCP-1 were described previously [14]. Plasmids expressing shRNAs targeting β-TrCP mRNAs were provided by J. Wade Harper and were expressed from modified versions of pSuperRetro: the target sequences are GAGAGAGAAGACUGUAAUA for β-TrCP-1, GCCCAUGUUGCAGCGGGAC for β-TrCP-2, and GUGGAAUUUGUGGAACAUC for both β-TrCP-1 and 2; a vector targeting *Renilla* GFP (rGFP) was used as a control [50],[51]. The proviral plasmid pNL4-3 was obtained from the National Institutes of Health (NIH) AIDS Research & Reference Reagent Program [52]. The pNL4-3 mutant ΔVpu (vpuDEL-1) and the pcDNA3.1-based plasmid expressing codon-optimized Vpu (pVphu) were provided by Klaus Strebel [19],[53]. Mutations were introduced into the coding sequence of the pVphu plasmid using the Stratagene QuickChange kit, and the presence of the desired mutations as well as the absence of unwanted mutations were verified by nucleotide sequencing. The plasmid expressing GFP (pCG-GFP) was provided by Jacek Skowronski [54]. A plasmid expressing IL-2 receptor α [Tac antigen; CD25 (pCDM8-Tac)] was provided by Juan Bonifacino [55]. The murine monoclonal antibody to BST-2/HM1.24/CD317 was a gift from Chugai Pharmaceutical Co., Kanagawa, Japan. For flow cytometry, an IgG2a antibody isotype control and a goat, anti-mouse IgG antibody conjugated to allophycocyanin (APC) was obtained from BioLegend (San Diego, CA); phycoerythrin (PE)-conjugated anti-CD25 was obtained from Becton-Dickinson. Rabbit antisera to HIV-1 Vpu and p17/p55 were obtained from the NIH AIDS Research & Reference Reagent Program. The murine monoclonal antibody to the α subunit of AP-2 was obtained from Affinity Bioreagents. The murine monoclonal antibody to HA was obtained from Covance. Secondary antibodies for immunofluorescence were obtained from Jackson ImmunoResearch (West Grove, PA). The vATPase inhibitor bafilomycin A1 was obtained from Sigma-Aldrich. The proteasome inhibitor MG-132 was obtained from Calbiochem.

### Cells and transfections

Unless specifically noted, the HeLa cells used in this study were clone P4.R5, which express both CD4 and CCR5 and obtained from Ned Landau; these cells are a derivative of clone P4 [56] and were maintained in DMEM plus 10% FBS, penicillin/streptomycin, and puromycin. Where noted, the CD4-negative HeLa clone Z24 was used; this line is a precursor to P4 cells and was provided by Chris Aiken. Cells were transfected using Lipofectamine2000 (Invitrogen) according to the manufacturer's instructions. For the experiments involving expression of ΔF-box β-TrCP, cells were transfected in wells of six-well plates using 4 µg total DNA: 2 µg of the empty vector control, the β-TrCP-expression vector, or the ΔF-box β-TrCP-expression vector; and 2 µg of the empty vector control, pVphu, or pNL4-3 or the ΔVpu proviral mutant. For experiments involving the expression of shRNAs, cells were first transfected in wells of twelve-well plates containing 1.6 µg total DNA, of which either all was the shRNA expression vector or 0.3 µg was pCG-GFP and the rest was the shRNA vector, then subsequently transfected with 0.32 µg each of pCDM8-Tac and pVphu. For experiments involving the expression of Vpu proteins containing substitutions in the DSGxxS motif or a termination codon at position 33 (Vpu-32), cells were transfected in wells of twelve-well plates containing 1.6 µg total DNA, of which 160 ng was pVphu; the remaining DNA was pCG-GFP and pcDNA3.1 for the flow cytometry assays, or the ΔVpu proviral mutant for the virion release assays. For the transfections combining the expression of wild type Vpu with Vpu-52/56N, 160 ng of pVphu and 1000 ng of pVphu-52/56N were used.

### Flow cytometry

For analysis of surface levels of BST-2, cells were stained before fixation in phosphate buffered saline (PBS) including sodium azide and 2% FBS at 4°C using an indirect method to detect BST-2: the HM1.24 murine monoclonal antibody (0.1 µg/ml) was followed by a goat anti-mouse IgG conjugated to APC. For the analysis of cells transfected sequentially to express shRNAs followed by Vpu and Tac antigen, the cells were stained first for BST-2 as just described, then blocked with normal mouse serum, and finally stained for Tac antigen using the PE-conjugated antibody to CD25. For the analysis of total cellular levels of BST-2 (intracellular staining), cells were fixed and permeabilized with the Cytofix/Cytoperm kit (BD Biosciences), followed by indirect staining as described above. All samples were analyzed by two- or three-color flow cytometry; gates for BST-2 were set using an antibody isotype control (IgG2a) as the primary antibody, gates for GFP and were set using non-GFP expressing cells, and gates for Tac were set using a PE-conjugated isotype control. Composite data profiles were created using FlowJo software (Tree Star, Inc.), as were MFI determinations of specific populations where indicated in the supplementary data.

### Virion-release assays

A p24 antigen capture ELISA (Perkin-Elmer) was used to determine the concentration of viral capsid protein in culture supernatants that were first clarified by centrifugation at 400 *g* as well as the concentration of capsid protein in detergent lysates (0.5% Triton-X-100 in PBS) of the adherent cells. The percentage of p24 capsid secreted into the culture media was determined as the concentration of p24 antigen in the supernatants divided by the concentration of p24 antigen in the total culture (supernatant plus cells) ×100.

### siRNA knockdowns

siRNAs to the μ subunits of AP-1, -2, and -3 were obtained from Dharmacon as 21-nucleotide duplexes with 3′dTdT overhangs. The following mRNA sequences were targeted: μ1, AAGGCAUCAAGUAUCGGAAGA-3; μ2, 5AAGUGGAUGCCUU UCGGGUCA; and μ3, AAGGAGAACAGUUCUUGCGGC; these siRNAs were validated previously [57]. The siCONTROL siRNA 1 (Dharmacon) was used as a non-targeting control. Cells were transfected with the siRNA duplexes using Lipofectamine 2000. Three days later, the cells were re-transfected with either pcDNA3.1 or pVphu, along with pCG-GFP, and then stained the following day for flow cytometry or immunofluorescence microscopy.

### Endocytosis assay

Cells were incubated with antibody to BST-2 at 4°C as described above except using PBS-FBS without azide for one hour, then washed, and incubated in DMEM with 10% fetal bovine serum for various times at 37°C to allow internalization of surface antigens. Internalization was stopped by the addition of ice-cold azide-containing PBS-FBS. The cells were then stained using a goat anti-mouse IgG conjugated to APC, fixed in formaldehyde, and analyzed by flow cytometry. For each condition (“no Vpu” or “+Vpu”), the mean fluorescence intensities (MFIs) of the “time 0” samples, which were never incubated at 37°C, were set at 100%.

### Immunofluorescence microscopy

Cells were stained for HIV-1 Vpu and AP-2 using the antibodies above after fixation in 3% formaldehyde and permeabilization using 0.1% NP40, both in PBS, as previously described [58]. Images were obtained using a spinning disc confocal fluorescence microscope fitted with a 100× objective (Olympus). For each field, a Z-series of images was collected, and the data were processed using a deconvolution algorithm (Slidebook software, Imaging Innovations, Inc). Cells were stained for surface BST-2 and HIV-1 Gag p17/p55 by fixation and staining for BST-2, followed by permeabilization as above and staining for p17/p55. For analysis of the relationship between cell surface BST-2 and Gag, images of single focal planes adjacent to the cover glass were deconvolved using a “no neighbors” algorithm (Slidebook). A Pearson correlation coefficient for the overlap between BST-2 and Gag was determined using at least 10 cells expressing either wild-type or Vpu-negative virus using Slidebook software. To analyze the relationship between the intensities of BST-2 and Gag at individual puncta on the plasma membrane, the intensities of over 100 puncta on the surface of seven cells each for wild-type and Vpu-negative virus were measured using Slidebook software. Composite multi-color images of single optical sections were assembled using Adobe Photoshop software.

### Western blots

Cellular samples were suspended in loading buffer containing SDS and dithiothreitol and boiled for 10 min. After resolution on a 12% denaturing polyacrylamide gels (Bio-Rad, Hercules, CA), the proteins were transferred to polyvinylidene difluoride membranes and blotted with the antibodies to Vpu, BST-2, or HA described above or with antibody to β-actin (Sigma). Detection was performed using a goat anti-mouse antibody linked to horseradish peroxidase (Bio-Rad) or a goat anti-rabbit antibody linked to horseradish peroxidase (Pierce), followed by development with enhanced chemiluminescence (GE Healthcare, Piscataway, NJ).

## Supporting Information

Figure S1 Quantitative effects of shRNAs targeting β-TrCP on the down-regulation of BST-2 by Vpu. (A) This experiment is an exact repeat of the one shown in Figure 1C. Cells (HeLa) were transfected with plasmids expressing shRNAs targeting either *Renilla* GFP (rGFP) as an irrelevant control, β-TrCP-1, β-TrCP-2, or both β-TrCP-1 and -2; in two cases these plasmids also expressed jellyfish GFP, for the others a separate plasmid expressing GFP was co-transfected. Two days later, the cells were re-transfected with an empty plasmid or a plasmid expressing Vpu, along with a plasmid expressing Tac antigen (IL-2 receptor α; CD25) as a transfection marker. The next day, the cells were stained for surface BST-2 and Tac, and then analyzed by three-color flow cytometry. Two-color dot plots are the BST-2 vs. Tac intensity of the individual GFP-positive cells. In each analysis, comparable regions of peak cell density for Tac-positive and BST-2 down-regulated cells were picked using the “auto-gating” tool of FlowJo software; the mean fluorescence intensity of each region is shown numerically within each plot. (B) The data from the above experiment, together with data derived similarly from the experiment shown in Figure 1C, were used to calculate the fold-down-regulation of BST-2 by Vpu in the presence of each shRNA. Error bars indicate the actual values from the two experiments; the average is graphed.
(1.09 MB TIF)

Figure S2 Inhibitory effect of ΔF-box β-TrCP on the release of virions from CD4-negative cells. The experiment was performed as described in the legend of Figure 2, except that CD4-negative HeLa cells (clone Z24) were used. The average values from two independent experiments are graphed; the error bars indicate the actual values obtained from each experiment.
(3.15 MB TIF)

Figure S3 Effect of Vpu on the cellular expression of BST-2 after enrichment of transfected cells by flow-sorting. The experiment was performed as described in the legend of Figure 4A, except that the transfected cells were physically sorted to enrich for GFP-positive (transfected) cells. (A) Immunoblot of the sorted cells for Vpu, BST-2, and actin. (B) Percentages of GFP-positive cells in the pre- and post-sorted samples. (C) Two-color flow cytometric data for the pre- and post-sorted samples.
(4.41 MB TIF)
