# Supplementary figures and images for: Vpu Antagonizes BST-2–Mediated Restriction of HIV-1 Release via β-TrCP and Endo-Lysosomal Trafficking
Source: PLoS Pathog. 2009 May 29;5(5):e1000450. doi: 10.1371/journal.ppat.1000450 (PMC2679223; doi:10.1371/journal.ppat.1000450)

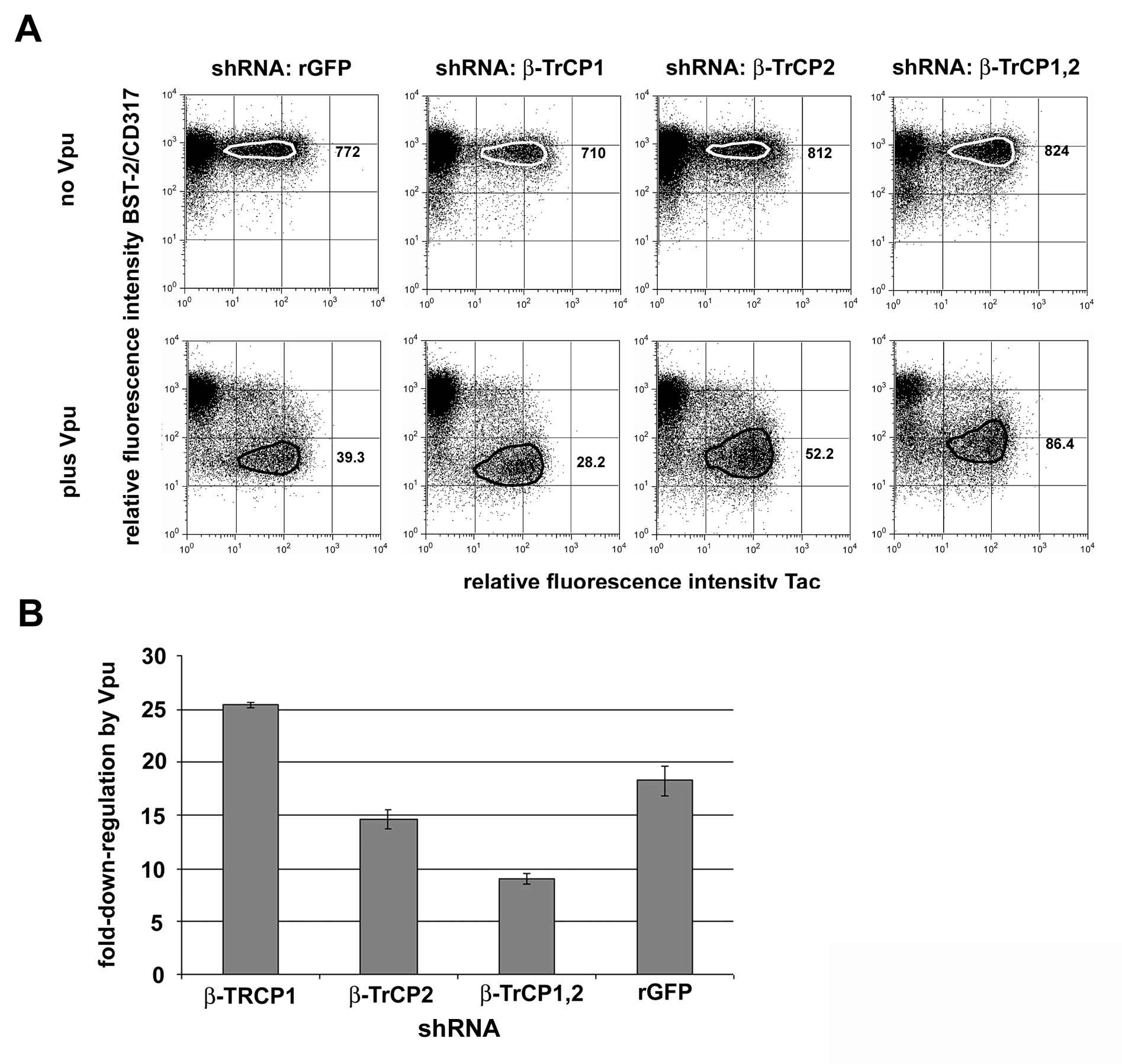

Supplement: Figure S1 — Quantitative effects of shRNAs targeting β-TrCP on the down-regulation of BST-2 by Vpu. (A) This experiment is an exact repeat of the one shown in Figure 1C. Cells (HeLa) were transfected with plasmids expressing shRNAs targeting either Renilla GFP (rGFP) as an irrelevant control, β-TrCP-1, β-TrCP-2, or both β-TrCP-1 and -2; in two cases these plasmids also expressed jellyfish GFP, for the others a separate plasmid expressing GFP was co-transfected. Two days later, the cells were re-transfected with an empty plasmid or a plasmid expressing Vpu, along with a plasmid expressing Tac antigen (IL-2 receptor α; CD25) as a transfection marker. The next day, the cells were stained for surface BST-2 and Tac, and then analyzed by three-color flow cytometry. Two-color dot plots are the BST-2 vs. Tac intensity of the individual GFP-positive cells. In each analysis, comparable regions of peak cell density for Tac-positive and BST-2 down-regulated cells were picked using the “auto-gating” tool of FlowJo software; the mean fluorescence intensity of each region is shown numerically within each plot. (B) The data from the above experiment, together with data derived similarly from the experiment shown in Figure 1C, were used to calculate the fold-down-regulation of BST-2 by Vpu in the presence of each shRNA. Error bars indicate the actual values from the two experiments; the average is graphed. (1.09 MB TIF) [file ppat.1000450.s001.tif]

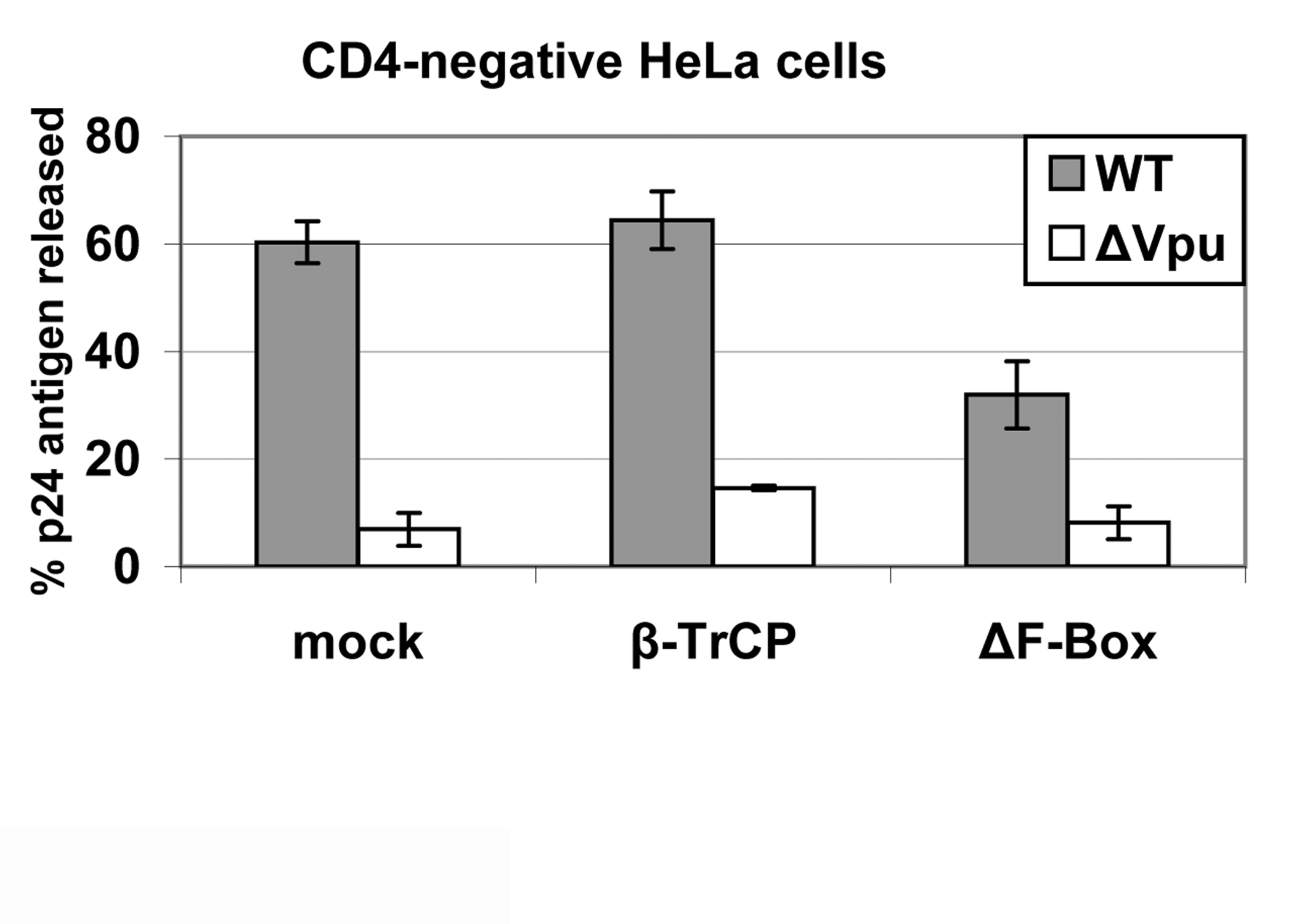

Supplement: Figure S2 — Inhibitory effect of ΔF-box β-TrCP on the release of virions from CD4-negative cells. The experiment was performed as described in the legend of Figure 2, except that CD4-negative HeLa cells (clone Z24) were used. The average values from two independent experiments are graphed; the error bars indicate the actual values obtained from each experiment. (3.15 MB TIF) [file ppat.1000450.s002.tif]

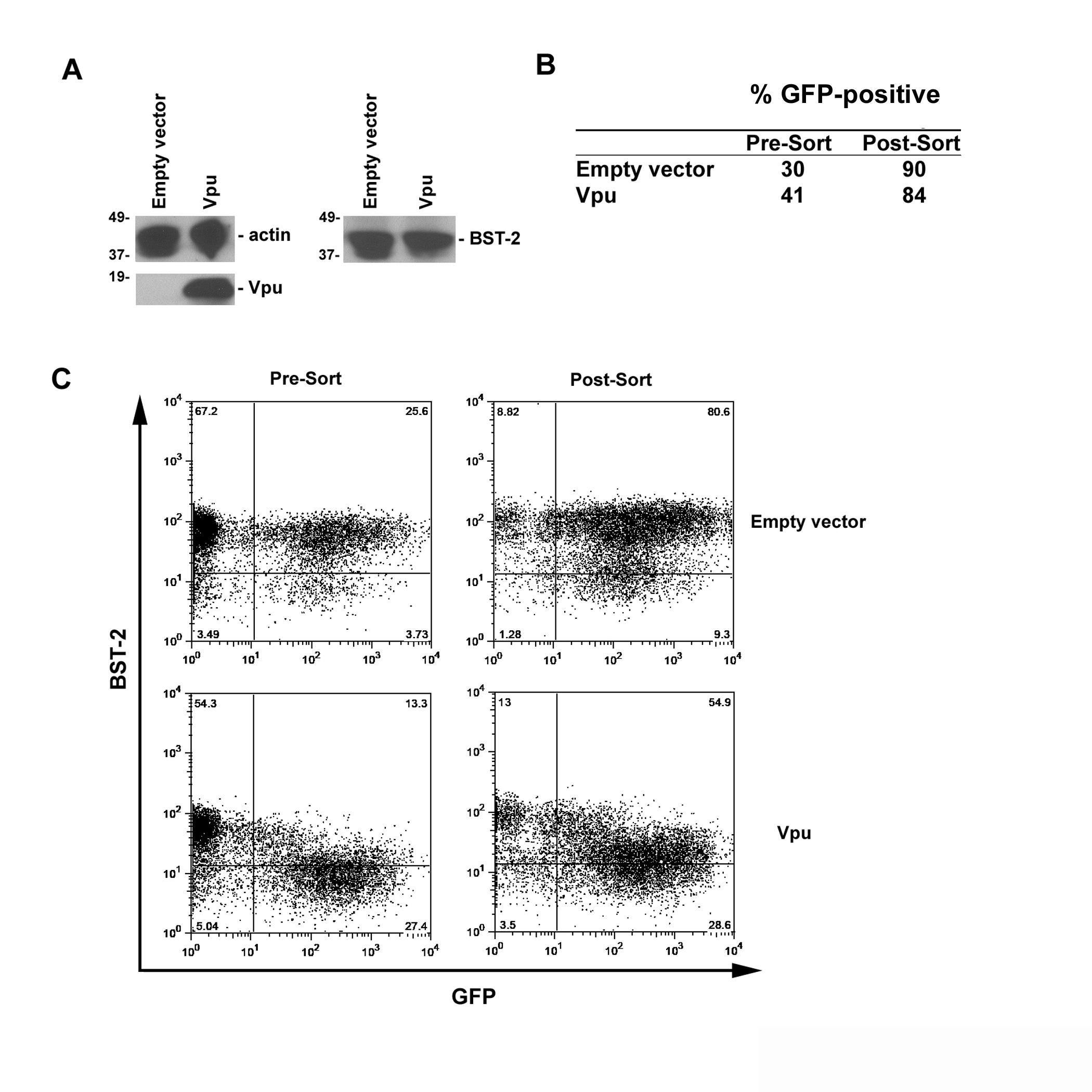

Supplement: Figure S3 — Effect of Vpu on the cellular expression of BST-2 after enrichment of transfected cells by flow-sorting. The experiment was performed as described in the legend of Figure 4A, except that the transfected cells were physically sorted to enrich for GFP-positive (transfected) cells. (A) Immunoblot of the sorted cells for Vpu, BST-2, and actin. (B) Percentages of GFP-positive cells in the pre- and post-sorted samples. (C) Two-color flow cytometric data for the pre- and post-sorted samples. (4.41 MB TIF) [file ppat.1000450.s003.tif]
